# Optimising the manufacturing of a β-Ti alloy produced via direct energy deposition using small dataset machine learning

**DOI:** 10.1038/s41598-024-57498-w

**Published:** 2024-03-23

**Authors:** Ryan Brooke, Dong Qiu, Tu Le, Mark A. Gibson, Duyao Zhang, Mark Easton

**Affiliations:** Centre for Additive Manufacturing, School of Engineering, RMIT University, Melbourne, VIC 3000 Australia

**Keywords:** Additive manufacturing, Direct energy deposition, Titanium alloys, Machine learning, Modelling, Materials science, Mechanical engineering

## Abstract

Successful additive manufacturing involves the optimisation of numerous process parameters that significantly influence product quality and manufacturing success. One commonly used criteria based on a collection of parameters is the global energy distribution (GED). This parameter encapsulates the energy input onto the surface of a build, and is a function of the laser power, laser scanning speed and laser spot size. This study uses machine learning to develop a model for predicting manufacturing layer height and grain size based on GED constituent process parameters. For both layer height and grain size, an artificial neural network (ANN) reduced error over the data set compared with multi linear regression. Layer height predictions using ANN achieved an R^2^ of 0.97 and a root mean square error (RMSE) of 0.03 mm, while grain size predictions resulted in an R^2^ of 0.85 and an RMSE of 9.68 μm. Grain refinement was observed when reducing laser power and increasing laser scanning speed. This observation was successfully replicated in another α + β Ti alloy. The findings and developed models show why reproducibility is difficult when solely considering GED, as each of the constituent parameters influence these individual responses to varying magnitudes.

## Introduction

Additive manufacturing (AM) allows computer aided designed models to be manufactured to a near-net-shape in a layer-by-layer fashion. Powder based direct energy deposition using laser beam (DED-LB) does this by focusing metal powder transported by carrier gas to a focal point to be met by a laser, which melts the powder to form layers of the part^1^. DED-LB has a higher deposition rate and build volumes when compared to other powder fed systems such as laser powder bed fusion (PBF-LB) and electron beam powder bed fusion (PBF-EB) and better feature resolution than wire-based systems such as wire arc additive manufacturing (WAAM)^2^. Numerous process parameters are controlled by the user leading to variability in print quality and mechanical properties. Process parameters such as laser power, laser scanning speed, laser spot size, powder feed flowrate, layer height, carrier gas pressure, hatch spacing, and hatching pattern will all influence the manufacturing process and thereby the quality of the component^1^. Material composition also introduces variability, with each material having different thermodynamic and radiance properties. The extensive list of processing variables leads to a difficult and material specific optimization process.

When considering the influence of process parameters on DED-LB, many researchers consider a version of energy input into the system to be a key research parameter^3–5^. The way in which this energy input is described can vary from linearly, to over a surface area or volumetrically. Global energy density (GED) is a measure of input energy over a surface area (J/mm^2^). In this research, GED, as defined in Eq. (1), is broken down into its constituent parameters to assess how each parameter individually influences the layer height and measured grain size on a β-titanium alloy:

1 $${\text{GED }} = \frac{{\text{P}}}{{{\text{vd}}}}$$

where *P* is laser power in Watts, v the laser scanning speed in mm/sec and *d* the spot size in mm of the laser. These three parameters were chosen as variable process parameters for this study. GED was chosen as nomenclature, as referenced by Wolff et al.^6^ becoming common in recent reviews^7,8^ but can also be referenced as effective energy density^1^, laser energy density (LED)^5^ and laser energy area density (EAD)^4^. Furthermore, it is worth noting that these process parameters do not encapsulate the full gamut of available variable processing parameters. However, these were chosen as common user set parameters for DED-LB.

The deposition rate will dictate the time to manufacture a component and it is a function of the laser scanning speed and layer height. The layer height is a user set process parameter. This controls how far the nozzle moves in the z-direction preceding each layer and is dictated by the amount of material deposited by the previous layer. Maintaining the correct working distance between the nozzle and the model is vital for achieving the desired focal point. An incorrect layer height setting can disrupt this balance, resulting in an improperly set focal point. This, in turn, introduces variability in the build and raises the risk of defects in the final product^9^. The layer height is a response to the process parameters, which is often found by the user in an iterative process. There is an opportunity to instead model the response of various process parameters on the layer height and thus create a predictive model for setting this.

The layer-by-layer manufacturing of DED-LB leads to cyclic heating of deposited layers. Some of the previous layer will be remelted by the preceding layer and by varying process parameters, the amount of energy in the form of heat accumulated within the work piece can be manipulated. Microstructure is a function of the solidification velocity and thermal gradient^10,11^, therefore by adjusting the processing parameters microstructural changes are expected. Liu et al.^5^ found that by increasing GED the grain size also increased for Inconel 718 when using DED-LB. Similarly, Donik et al.^12^ determined that by increasing energy density, grain size also increased for AISI 316L manufactured using PBF-LB. However, it is unknown from this research if the parameters which make up GED all influence the outcome similarly. As grain refinement is ubiquitous with increased mechanical properties^13^, it is beneficial to understand which process parameters influence the microstructure most significantly.

In the context of using GED as a parameter in AM, a notable challenge emerges. Different combinations of process parameters can result in the same GED. For example, in reference to the calculation of GED in Eq. (1), an increase in scanning speed can be offset by a proportional increase in laser power to maintain a constant GED. These differences in processing conditions are critical, as they influence the melt pool dynamics and subsequently the microstructure and solidification characteristics of the material. Although GED is used extensively, it is becoming increasingly evident that a more nuanced approach, considering individual process parameters separately, can lead to more accurate and insightful results.

By varying the process parameters with an orthogonal central composite design (CCD) design of experiments (DOE)^14^ a process map for a chosen alloy was created. CCD DOE provides a good platform for experimentation and response collection over a chosen parameter space. It is an efficient method of experimentation and can provide response insight from fewer experimental runs. Orthogonal CCD is centrally accurate and allows estimation of the curvature of the responses, which is not available from the often used alternative Box-Behnke design^15^. The laser power, laser scanning speed and laser spot size were adjusted over the chosen process ranges to measure the response on the layer height and the resulting average grain size. Using the measured response, machine learning models were created using multi linear regression (MLR) and an artificial neural network (ANN) to further understand how each parameter acts on the measured responses.

Modelling was undertaken using MLR and an ANN and their accuracy compared. The benefit of MLR is a response equation where the magnitude of each parameters effect on the response can be assessed. The ‘black box’ nature of ANNs provides less clarity on the influence of each parameter on the response; however, it can provide an accurate predictive tool using any available data. The methods were used in tandem and assessed using the response data and their accuracy compared.

The use of machine learning (ML) for AM process parameter optimization has shown some success. Wang et al.^16^ was able to use ML techniques to optimize two PBF-EB process parameters, using data from 56 samples for optimized responses of ultimate tensile strength, relative density and top surface finish. However, this methodology requires comparatively high amounts of labour and is time intensive, meaning that there is less inclination for industrial users to replicate it. Lu et al.^17^ produced accurate ML models for deposition height of a single track deposition using DED-LB, it is unknown if these models translated into bulk build deposition layer height.

The purpose of this research is to use DOE with ML techniques to demonstrate a material specific modelling and predictive method with optimization utility. The results also indicate significant control over the solidification process leading to variation in produced grain size. A powerful prediction model is also produced, allowing the layer height setting to be set with less labour intensive means. Due to the complexity in the DED-LB process and material parameters, time and cost-efficient experiments can greatly support the further uptake of DED-LB manufacturing. By extension, this modelling process is expected to be applicable for other powder-based AM techniques, responses, process parameters and materials.

## Methodology

Ti–10Fe (nominal wt%) (powders supplied by TLS Technik) were mixed from elemental 50–100 μm diameter spherical powders and was blended for 2 h using a Turbula Shaker to ensure its homogeneity. This composition was selected for its fully β-Ti grain microstructure for ease in grain size measurement.

DED-LB was performed using the Trumpf TruLaser Cell 7020 with a continuous laser configuration. Process parameters were chosen over ranges regularly used for Ti-alloys and which produced stable single tracks from testing. A single track was deemed stable when it was consistent, even and bonded to the substrate, without splattering or balling. Once the process parameter ranges were chosen and the experimental parameters found through the CCD method, 5 initial layers of 10 × 10 mm were printed at 0.2 mm layer height, to find an initial approximate layer height. The total build height was measured, and the corresponding initial approximate layer height to be used for each experimental run was found. 10 × 10 × 10 mm^3^ cubes were printed onto a CP-Ti substrate with the CCD dictated process parameters and initially measured layer height. Other process parameters which were kept constant were powder flowrate (2.8 g/min), inert carrier gas flow rate (16l/min), inter-layer dwell time (20 s), hatch spacing (30% overlap) and hatch pattern (rotating 90° each layer). From 3 variables and chosen maximum and minimum process values, 16 experiments are dictated, 2 of which are central value experiments. The chosen maximum and minimum are dimensionless at − 1 and + 1 and the experimental range is extended to − 1.287 and + 1.287 axial ‘star points’. The axial ‘star points’ are calculated in Eq. (2) as:

2 $$\alpha = \left( {\frac{{\sqrt {N*f} - f}}{2}} \right)^{1/2}$$

where, N (= 16) is the number of experiments and f (= 8) is the number of trails of a factorial experiment. This creates an orthogonal CCD^14,15^. A further 9 experiments were run as validation for the produced models. The 25 experimental runs with variable laser power, laser scanning speed and spot size are listed in Table 1.

**Table 1:** Dimensioned experimental process parameters settings and the corresponding GED.

Experimental run	Input parameters			
	laser power (W)	Scanning speed (mm/min)	Spot size (mm)	GED (J/mm^2^)
1	600	600	1.2	50
2	900	600	1.2	75
3	600	1200	1.2	25
4	900	1200	1.2	38
5	600	600	1.8	33
6	900	600	1.8	50
7	600	1200	1.8	17
8	900	1200	1.8	25
9	557	900	1.5	25
10	943	900	1.5	42
11	750	514	1.5	58
12	750	1286	1.5	23
13	750	900	1.11	45
14	750	900	1.89	27
15	750	900	1.5	33
16	750	900	1.5	33
*17*	*800*	*800*	*1.5*	*40*
*18*	*900*	*900*	*1.5*	*40*
*19*	*700*	*700*	*1.2*	*50*
*20*	*750*	*900*	*1.5*	*33*
*21*	*600*	*800*	*1.2*	*38*
*22*	*800*	*800*	*1.2*	*50*
*23*	*750*	*1000*	*1.5*	*30*
*24*	*750*	*1000*	*1.8*	*25*
*25*	*700*	*500*	*1.5*	*56*

1–16 are the DOE planned experiments and 17–25 the validation experiments, as indicated by the italic underlined values.

**Figure 1:**
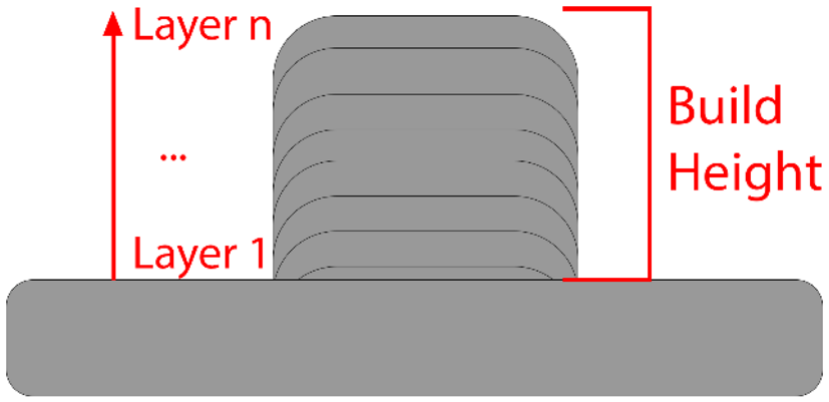
The measurement of layer height calculated from the build height divided by the number of layers built.

The 25 produced cube heights were measured, and the number of layers recorded as indicated in Fig. 1. The layer height was calculated as the build height divided by the number of layers. The cubes were removed from the substrate and cut in half. One half was mounted and polished before etching with Kroll’s reagent to reveal the β grain boundaries. The etched specimens were imaged using a Leica DM2500 optical microscope. Grain size was measured from the collected optical images using the linear intercept method until sufficient relative accuracy (RA < 10%) was achieved as per ASTM E112-113. For further validation, electron backscatter diffraction (EBSD) was collected at 20 kV and 20 nA, using a Jeol 7200F scanning electron microscope and Aztec software. Grain size was measured using Aztec Crystal software. Two EBSD grain size measurements were taken for the maximum and minimum optically measured grain sizes and were found to be within 11 μm and 6 μm respectively which was deemed within tolerability.

Each response was modelled using MLR and an ANN with 2 nodes in the single hidden layer using the sigmoidal activation function. The ANN used the Bayesian Regularization process as it has been shown to be robust with smaller data sets and not prone to overfit^18^. These were compared for accuracy of the produced models and validated against 9 experimental runs. In this paper, all models were trained using experiments 1–16, which were defined by the orthogonal CCD method, and then validated using experiments 17–25. Once validated, the models were retrained using all the data available for the presentation of the surface plots. The comparison of accuracy and goodness of fit of the models were made using the coefficient of determination (R^2^) shown in Eq. (3), root mean square error (RMSE) as in Eq. (4) and mean absolute percentage error (MAPE) as in Eq. 5^19,20^.

3 $${\text{R}}^{{2}} { = }\left( {\frac{{\mathop \sum \nolimits_{{\text{i = 1}}}^{{\text{N}}} \left( {{\text{y}}_{{\text{i}}} {\text{ - y}}_{{\text{a}}} } \right)\left( {\tilde{y}_{i} - \tilde{y}_{a} } \right)}}{{\sqrt {\mathop \sum \nolimits_{{\text{i = 1}}}^{{\text{N}}} \left( {{\text{y}}_{{\text{i}}} {\text{ - y}}_{{\text{a}}} } \right)^{{2}} } \sqrt {\mathop \sum \nolimits_{{\text{i = 1}}}^{{\text{N}}} \left( {\tilde{y}_{i} - \tilde{y}_{a} } \right)^{{2}} } }}} \right)^{{2}}$$

4 $${\text{RMSE = }}\sqrt {\frac{{1}}{{\text{N}}}\mathop \sum \limits_{{\text{i = 1}}}^{{\text{N}}} \left( {{\text{y}}_{{\text{i}}} { - }\tilde{y}_{{\text{i}}} } \right)^{{2}} }$$

5 $$MAPE = \frac{1}{N}\mathop \sum \limits_{i = 1}^{N} \left| {\frac{{\left| {y_{i} - \tilde{y}_{i} } \right|}}{{y_{i} }}} \right| \times 100$$

where N is the total number of experiments, y_i_ the experimental output value, ỹ_i_ the model predicated value (where i is the experimental run), y_a_ the mean experimental value and ỹ_a_ the mean model predicted value.

## Results and discussion

The measured results for the 25 experimental runs are listed in Table 2. The responses collected from each cube sample were the average grain size, and the manufacturing layer height.

**Table 2:** Measured responses of grain size and layer height for the 25 experimental runs listed in Table 1.

Experimental run	Grain size (μm)		Layer height (mm)
1	91.4	± 5.1	0.97
2	116.4	± 7.2	0.97
3	47.9	± 0.5	0.45
4	97.2	± 3.4	0.45
5	58.3	± 3.1	0.66
6	133.4	± 5.3	0.70
7	37.9	± 2.6	0.32
8	77.8	± 4.8	0.32
9	41.5	± 0.3	0.47
10	110.3	± 6.2	0.48
11	91.6	± 1.2	0.88
12	57.9	± 3.3	0.45
13	69.8	± 5.3	0.69
14	51.4	± 4.0	0.41
15	72.8	± 6.7	0.53
16	72.5	± 5.1	0.52
*17*	*79.7*	± *1.4*	*0.57*
*18*	*110.0*	± *3.7*	*0.46*
*19*	*71.0*	± *4.9*	*0.71*
*20*	*69.8*	± *5.6*	*0.48*
*21*	*63.9*	± *1.8*	*0.70*
*22*	*109.2*	± *9.6*	*0.70*
*23*	*62.9*	± *0.9*	*0.44*
*24*	*71.7*	± *4.4*	*0.37*
*25*	*103.0*	± *8.8*	*0.91*

Experiments 17–25 being validation experiments, as indicated by the italic underlined values.

Experimental runs 1, 6, 19, 22 all have the same GED input value of 50 J/mm^2^. For these experimental runs the average grain size varies from 71 to 133 μm and the layer height varies from 0.7 to 0.97 mm. It is evident from these results that using GED as a guiding process parameter will make reproducibility of results difficult with respect to the grain size and layer height.

### Layer height

**Figure 2:**
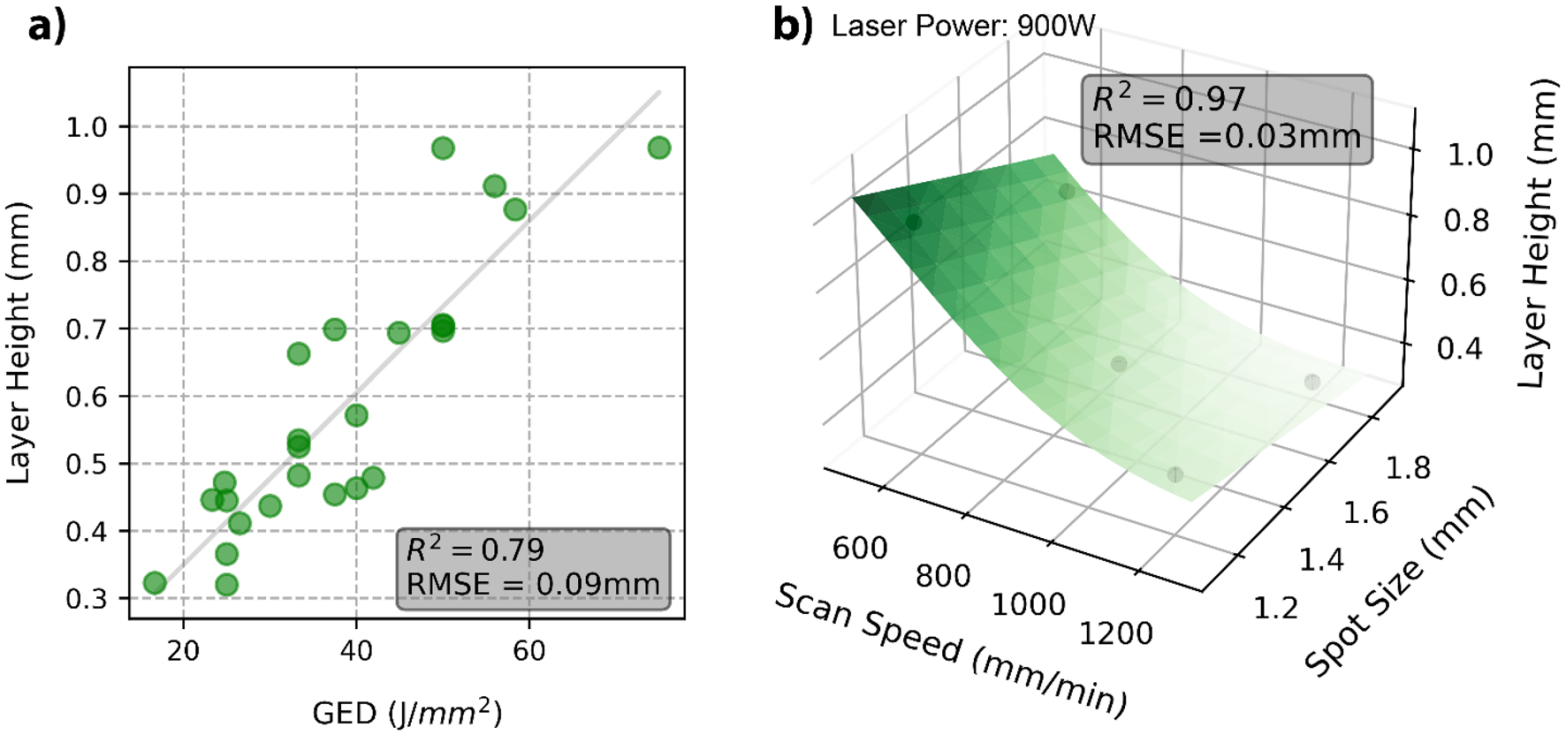
(**a**) The relationship between the GED and the measured layer height with a fitted ordinary least squares linear regression and corresponding R^2^ and RMSE. (**b**) Layer height response at 900 W Laser Power, modelled using an ANN. R^2^ and RMSE correspond to the full dataset trained model. The black data points are the experimental data points with a 900 W laser power.

Measured layer heights ranged from 0.32 to 0.97 mm for the 25 samples produced with the various process parameters. Figure 2a displays a rough linear relationship between GED and the measured layer height. Liu et al.^5^ and Shim et al.^21^ found similarly that increasing GED led to an increasing layer height when manufacturing using DED-LB. An ordinary least square linear regression fit on Fig. 2a produces a R^2^ value and data set RMSE of 0.79 and 0.09 mm respectively. Figure 2a also shows how the same GED can lead to variation in the produced layer height, therefore with a better fitting model, more accurate prediction of layer height could be achieved.

The layer height response to the three separate process parameters was best fit by the ANN, as shown by the fit and error data in Table 3. Upon training with the initial 16 data points, the model fits most accurately to the ANN with a R^2^ and RMSE of 0.98 and 0.03 mm respectively. The 9 data points of the test data set shows validation of the model, with only a slight increase in RMSE to 0.05 mm. Upon validation and retraining with the full data set on the ANN, the R^2^ and RMSE were 0.97 and 0.03 mm respectively. The lowest mean absolute percentage error was achieved by the ANN at 5.10%, indicating the greatest prediction accuracy. The MLR model retrained on the full dataset did also show good fit with a R^2^ and RMSE of 0.88 and 0.07 mm respectively. Although the ANN produced less error, the MLR model equation, shown in Eq. (6) quantifies which of the targeted parameters have the strongest influence on the response. Laser power has only a minor influence on the layer height response over the process parameter range for this material. As laser speed and spot size increase, the layer height decreases, with laser speed having almost double the effect. Slower laser scanning speed increases the layer height, which is the result of more time for the powders to be delivered to the melt pool and to interact with the laser, melting more material. The layer height is a function of the flowrate of powder, the area of this interaction (the spot size) and the time of this interaction (laser scanning speed). As the process parameters were tested for stability using single tracks, the laser power parameter range is sufficient to melt the incident powder, this may not be the case if the powder flowrate is increased.

**Table 3:** R^2^, RMSE and MAPE for layer height models using ordinary least squares on GED (OLS-GED), MLR and ANN on the individual parameters.

	OLS-GED	MLR	ANN
R^2^-training		0.90	0.98
R^2^-test		0.91	0.96
R^2^-full dataset trained	0.79	0.88	0.97
RMSE-training		0.06 mm	0.03 mm
RMSE-test		0.08 mm	0.05 mm
RMSE-full dataset trained	0.09 mm	0.07 mm	0.03 mm
MAPE-full dataset trained	12.16%	10.28%	5.10%

R^2^, RMSE and MAPE calculated by Eqs. (3), (4) and (5) respectively.

The MLR model equation for layer height using scaled laser power (P_s_), laser scanning speed (v_s_) and spot size (d_s_) (scaled process parameters are centered on the mean and scaled to a standard deviation of 1) is given by:

6 $${\text{Layer}} \,{\text{Height}} = 0.58436 - 0.00372 \times P_{s} - 0.15469 \times v_{s} - 0.07526 \times d_{s}$$

In Fig. 2b the ANN model is represented as a response surface at 900 W laser power. As laser power has only a small influence on the layer height as shown in Eq. (6), surface plots at 600 W or 750 W are similar. A distinct benefit of the ANN is being able to retrain the model as more data becomes available and for prediction purposes, this should increase the accuracy of the model. An accuracy comparison of the linear model and the ANN can be made by comparing the R^2^ and RMSE values. The linear model has an R^2^ of 0.79 and RMSE of 0.09 mm compared with 0.97 and 0.03 mm for the ANN. This information indicates the much more powerful modelling of the ANN with the three separate process parameters when compared with the linear modelling of a single amalgamated GED process parameter.

**Figure 3:**
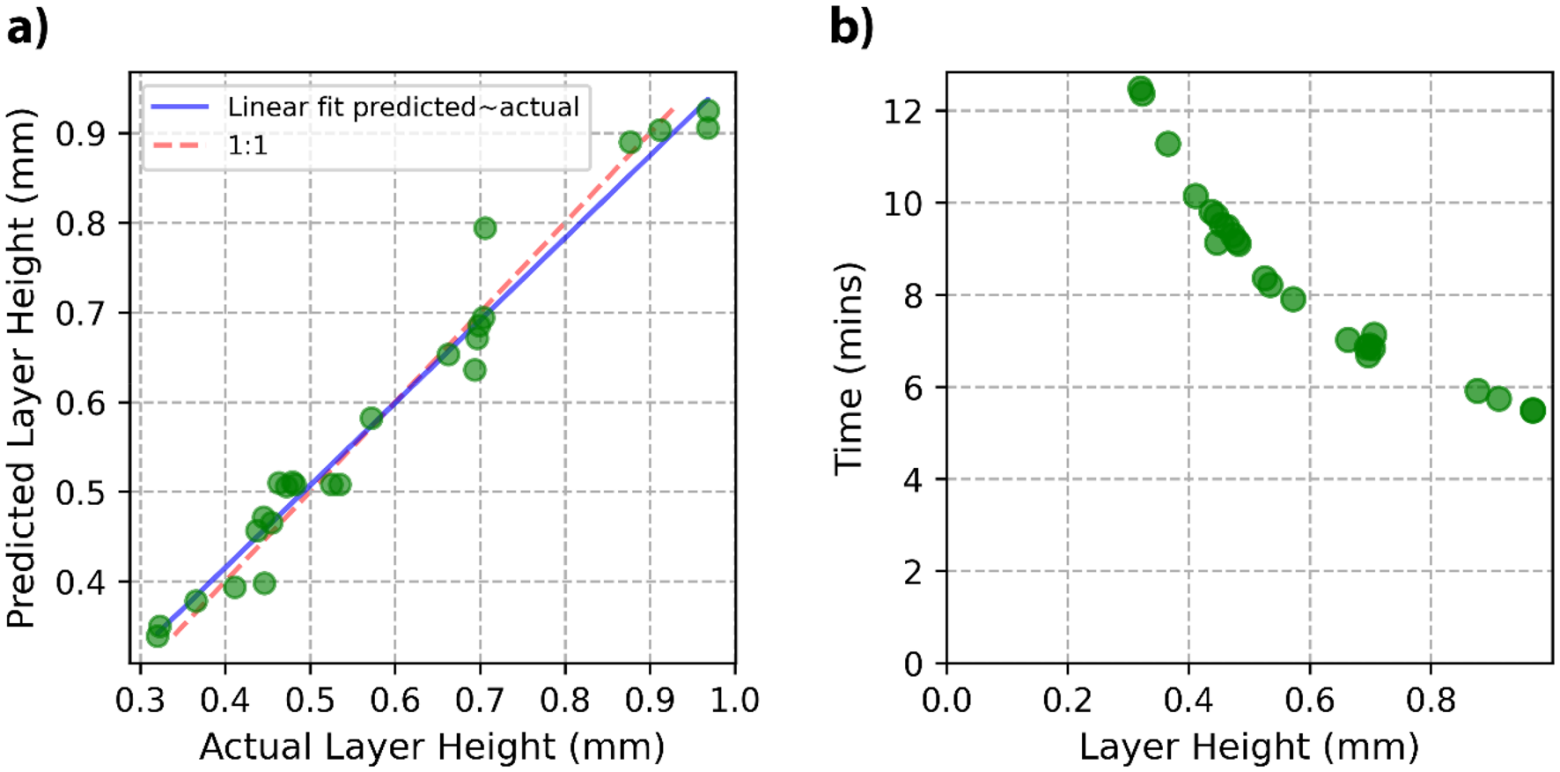
(**a**) Layer height ANN actual measured vs. the predicted layer height. (**b**) Time to produce a 10 × 10 × 10 mm^3^ cube calculated using the experimentally measured layer height and scanning speed.

As the ANN trained with 16 data points was validated over the 9 data points, it was retrained using the full data set for an accurate prediction model. The prediction of layer height produced a maximum error of 0.09 mm for experimental settings #19. After 10 layers, this would equate to less than 1mm in build error. Although this would still lead to less stable and predictable production it is unlikely to be catastrophic. As captured in Fig. 3a, the ability of the ANN to predict the required layer height for the process parameters is significant. Therefore, the ANN predictive model is a powerful tool in reducing the setup time of manufacturing this material.

The layer height is a large contributor to the deposition rate. The smallest experimentally measured layer height of 0.32 mm will take approximately 12.5 min to produce a 10 × 10 × 10 mm^3^ cube, compared with 5.5 min for the layer height of 0.97 mm as shown in Fig. 3b. Increasing layer height reduces the time for cube production. Increased layer height can be achieved by decreasing the laser scanning speed and the laser spot size, as shown by Eq. (6). This provides a mechanism in which production time can be decreased through the judicious selection of the process parameters.

**Figure 4:**
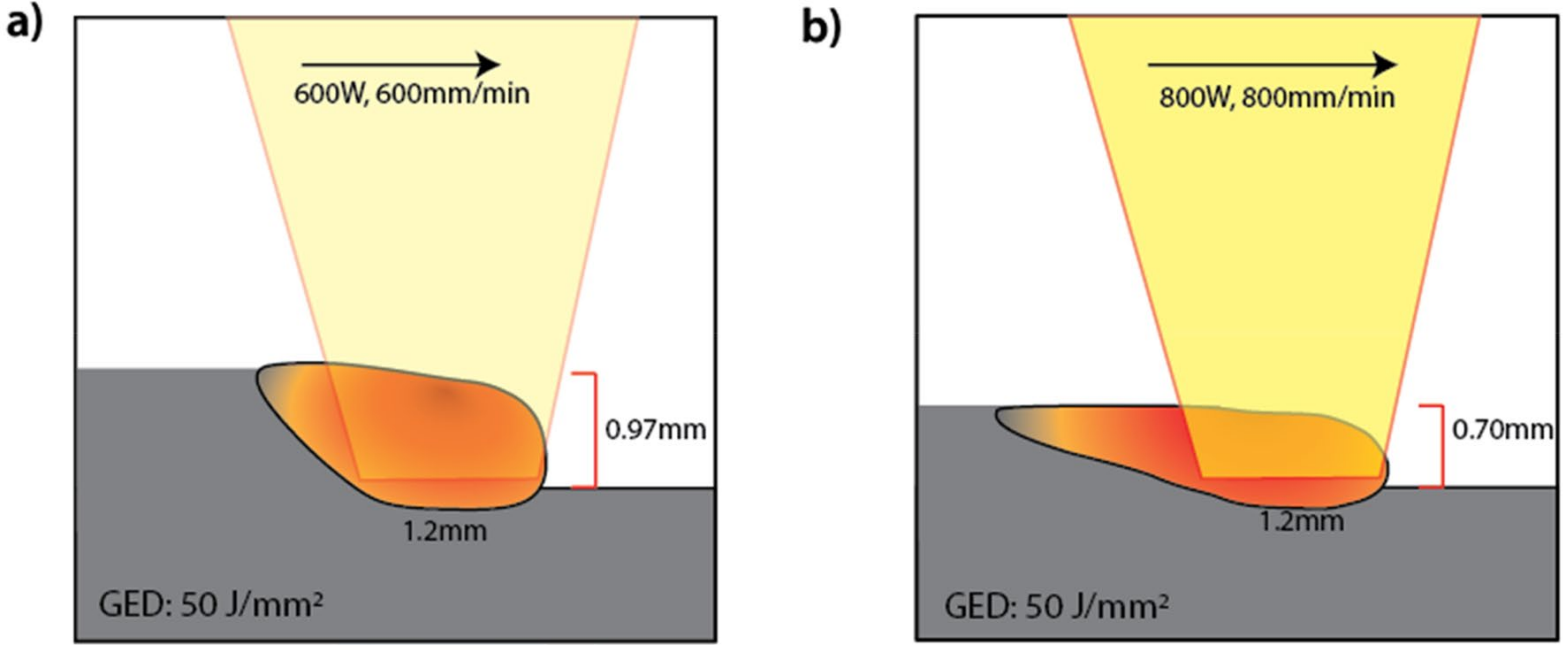
(**a**) Schematic diagram of experimental run #1 with a GED of 50 J/mm^2^ (laser power of 600 W, scanning speed of 600 mm/min and spot size of 1.2 mm) producing a layer height of 0.97 mm. (**b**) Schematic diagram of experimental run #22 with a GED of 50 J/mm^2^ (laser power of 800 W, scanning speed of 800 mm/min and spot size of 1.2 mm) producing a layer height of 0.70 mm.

GED overlooks the impact of the scanning speed on the incident powder. Keeping a constant powder flow rate (g/min) means that as the scanning speed increases—from 600 to 800 mm/min as in the schematics for experimental run #1 and #22 of Fig. 4a,b—the effective powder deposition rate linearly decreases, from 4.7 to 3.5 g/m, respectively. This can explain the reduction in layer height from 0.97 to 0.70 mm with increasing speed reducing the incident powder while maintaining the same GED input. The MLR equation shown by Eq. (6) supports the large influence laser scanning speed has on layer height. Changing laser power could also influence the effective powder usage, highlighting the complexity which is not considered by amalgamating the individual parameters into GED. The resulting change in the melt pool dimensions will also change the peak temperature and thermal gradient within the melt pool, changing solidification conditions. By modelling the parameters individually, greater insight into their influence is achieved.

### Grain size

**Figure 5:**
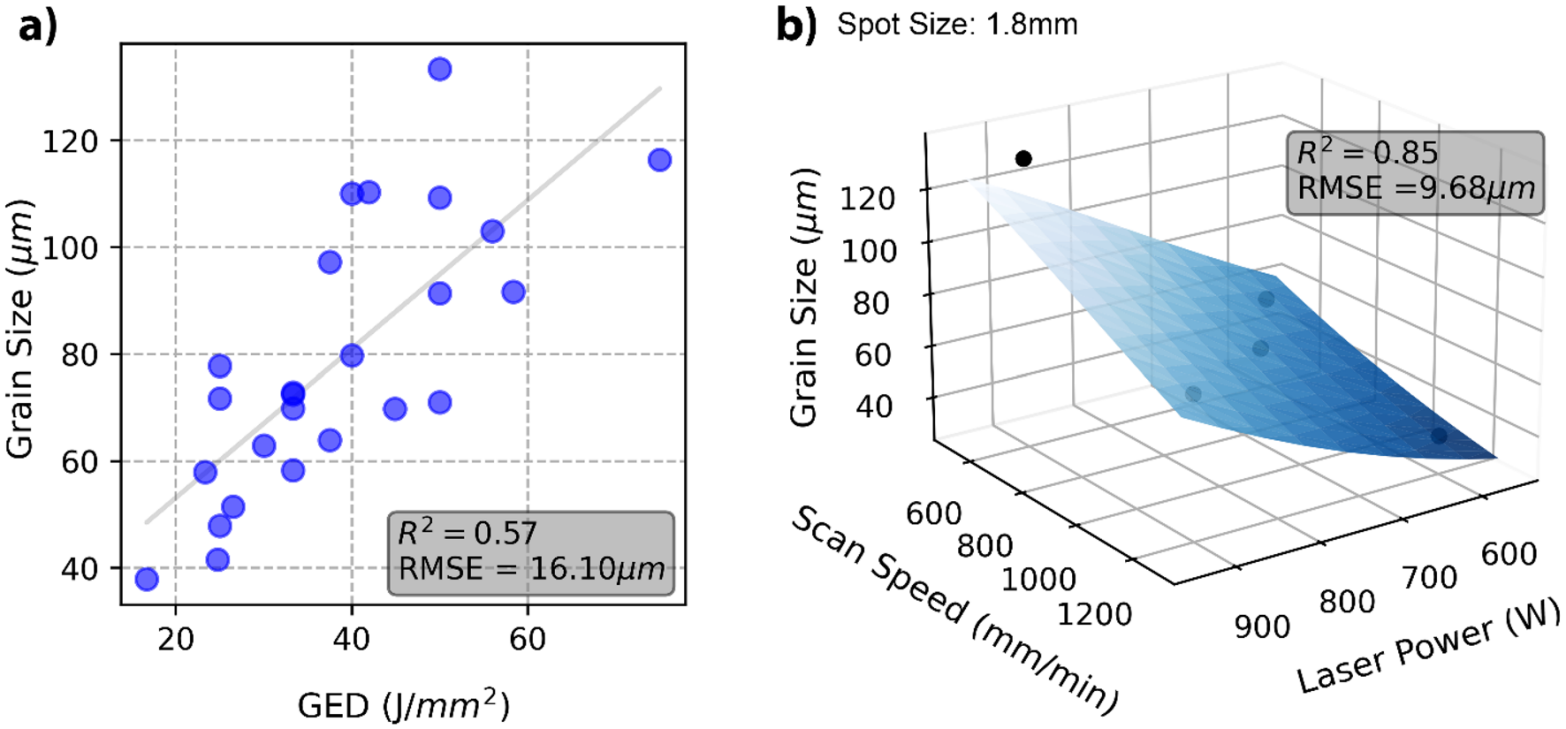
(**a**) GED vs average grain size with a fitted ordinary least squares linear regression and corresponding R^2^ and RMSE. (**b**) The average grain size as a response to changing laser power and scanning speed at 1.8mm spot size, modelled using an ANN. R^2^ and RMSE correspond to the full dataset trained model.. The black data points are the experimental data points with a 1.8mm spot size.

GED and average grain size do share a rough linear relationship as indicated in Fig. 5a Liu et al.^5^ found similarly that as GED increased the average grain diameter also increased.

**Figure 6:**
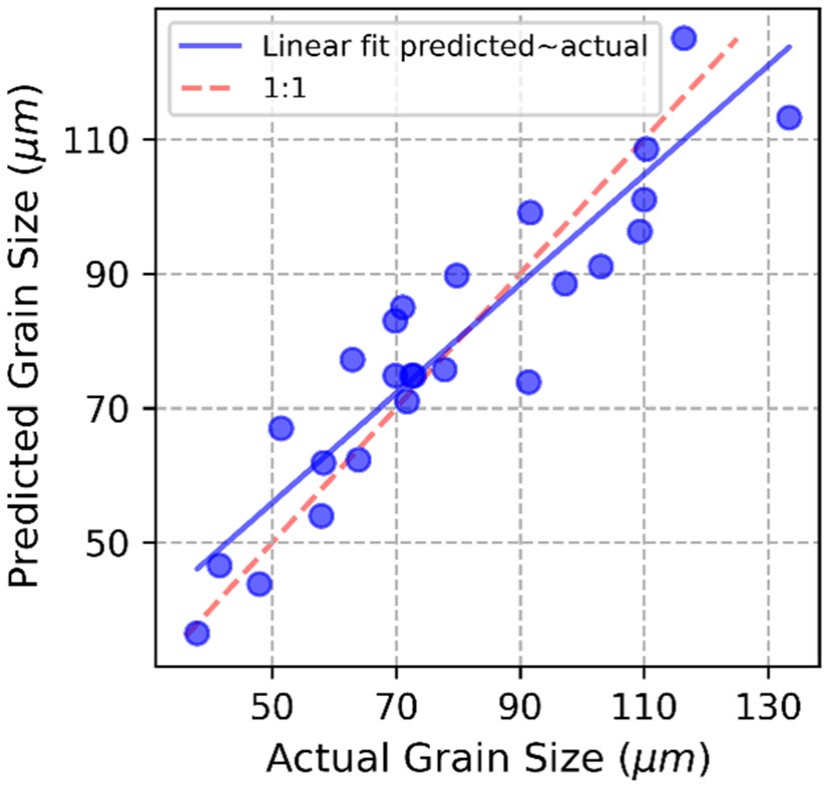
Grain size ANN actual measured vs. the predicted layer height.

The MLR and ANN models show similar accuracy, as shown in Table 4. Upon validation and retraining with the full data set the MLR and ANN showed a R^2^ of 0.83 and 0.85 respectively. The RMSE for the retrained MLR and ANN were 10.22 µm and 9.68 µm respectively. Both models produce an accurate fit, but the ANN shows marginally less error overall. Figure 6 shows the actual grain size vs the ANN predicted grain size plot, with the errors randomly distributed around the linear fit, indicating a well-fitting model. Both these models show greater accuracy when compared with a linear relationship between GED and the average grain size, which produced an R^2^ and RMSE of 0.57 and 16.1 μm, respectively. The lowest mean absolute percentage error was achieved by the ANN at 10.10%, indicating the greatest prediction accuracy.

**Table 4:** R^2^, RMSE and MAPE for grain size models using ordinary least squares on GED (OLS-GED), MLR and ANN on the individual parameters.

	OLS-GED	MLR	ANN
R^2^-training		0.87	0.87
R^2^-test		0.72	0.73
R^2^-full dataset trained	0.57	0.83	0.85
RMSE-training		9.96 µm	9.77 µm
RMSE-test		10.84 µm	10.76 µm
RMSE-full dataset trained	16.10 µm	10.22 µm	9.68 µm
MAPE-full dataset trained	16.76%	11.41%	10.10%

R^2^, RMSE and MAPE calculated by Eqs. (3), (4) and (5) respectively.

The MLR model for the average grain size using scaled laser power (P_s_), scanning speed (v_s_) and spot size (d_s_) (scaled process parameters are centered on the mean and scaled to a standard deviation of 1) is given by:

7 $${\text{Grain Size}} = 78.77 + 18.64 \times P_{s} - 12.87 \times v_{s} - 4.78 \times d_{s}$$

The MLR model given in Eq. (7) shows the greatest influence on the average grain size is the laser power followed by the scanning speed. As the laser power increases, so does the average grain size, and increasing the laser scanning speed will decrease the grain size. The spot size plays a much more minor role in the response on grain size over the range investigated, however, increasing the spot size tends to reduce the average grain size. Figure 5b shows the response at 1.8 mm spot size, which produces a smaller grain size compared with the 1.5 mm and 1.2 mm spot sizes. This response surface is a visualisation of the ANN prediction, which produced the least error. The response surface from the ANN shows the same trends as the MLR model, with laser power and scanning speed having a linear influence on the average grain size but to different magnitudes.

**Figure 7:**
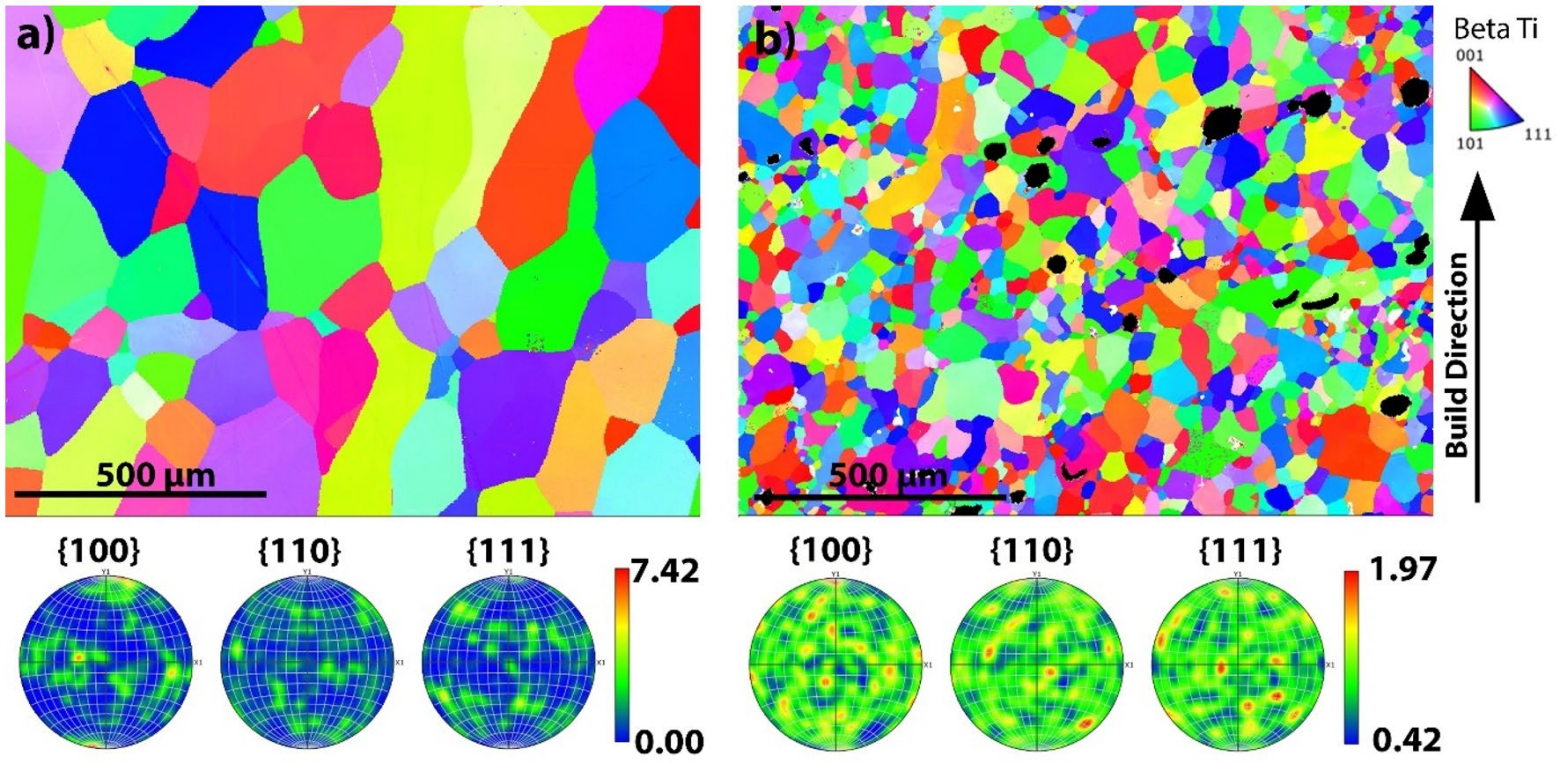
EBSD of (**a**) Experimental run #6 with the largest measured grain size of 133 μm. (**b**) Experimental run #7 with the smallest measured grain size of 38 μm.

The maximum and minimum measured grain sizes were 133μm and 38μm as depicted in Fig. 7a and b, respectively. Increasing GED, thereby increasing the heat input into the system leads to β-grain coarsening in the Ti–10Fe system. However, it is once again too simplistic to model the response using GED, as the process parameters influence the response to varying degrees. Spot size has a signifcantly less impact on the average grain size according to the MLR and ANN models compared to the laser power and scan speed parameters. These results show in order to reduce grain size it is more effiective to reduce laser power and increase laser scanning speed.

Other mechanisms may also be influencing grain refinement. Wang et al.^22^ characterises the grain refinement of TC11 Ti alloy due to an increase in powder mass flow rate, leading to unmelted powders nucleating β grains. This would lead to partially melted particles within the melt pool during deposition. There are some indications in Fig. 7b of Ti powder which has not been fully mixed into the greater melt, with some α-phase in the final microstructure obviously not in solution with Fe (shown as black spots), therefore it appears to only have been partially melted. Interestingly, this is at the faster scanning speed, which would indicate a lower powder flowrate per unit area, contrary to ref^22^, although laser power was also varied, again highlighting the complexity of the process and why multi variable optimization methodologies are important. It could be possible partially unmelted powders are responsible for some of the grain refinement, however they do not appear to be the nucleation sites for most of the refined grains, therefore it would appear the thermal characteristics of the melt pool and rapid solidification play a greater role. Figure 7a. depicts experimental run #6 which had the greatest observed grain size and no partially unmelted powders, it would appear the slower scanning speed allows greater mixing of the melt pool.

**Figure 8:**
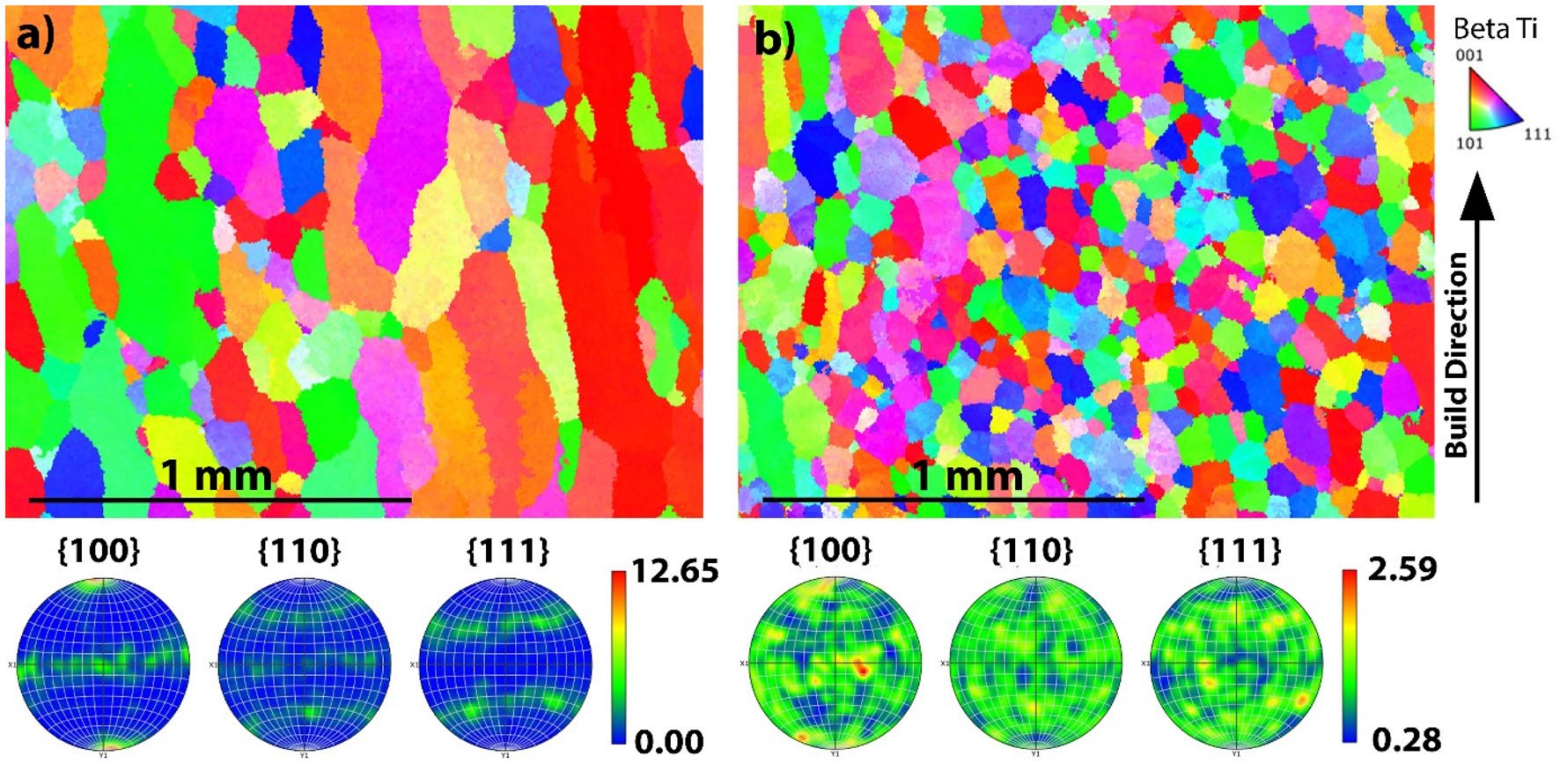
EBSD and parent grain analysis of (**a**) Quaternary Ti alloy produced via DED-LB using 800 W laser power, 800 mm/min laser scanning speed and a 1.5 mm spot size and (**b**) the same alloy produced with a laser power of 600W and a laser scanning speed of 950 mm/min.

The findings show the most effective parameters to induce grain refinement were a reduction in laser power and an increase in scanning speed. This was applied to a quaternary Ti–Cu–Fe–Al α + β alloy as imaged in Fig. 8a,b. The same alloy was produced with a reduction in laser power from 800 to 600W and an increase in scanning speed from 800 to 950 mm/min. The other parameters were kept constant (powder flowrate (1.8 g/min), inert carrier gas flow rate (16l/min), inter-layer dwell time (20 s), hatch spacing (30% overlap) and hatch pattern (rotating 90° between each layer), although there was a reduction in layer height from 0.38 to 0.25 mm corresponding to the changed parameters. The resulting prior-β grain refinement from 119 to 68 μm is substantial. Using a Hall–Petch equation to model the yield strength associated with grain size, $\Delta {\upsigma }_{{{\text{Hp}}}} {\text{ = 792 d}}^{{{ - }{0}{\text{.5}}}}$, as adopted from Hyun et al.^23^, the reduction in grain size from 119μm to 68μm will increase yield strength by 23 MPa. The grain refinement also coincides with a more complete columnar to equiaxed transition, with an increase in area made up of equiaxed grains (grains with an aspect ratio < 2) from 27.8 to 71.7%. Further to the refinement in the grains, the crystallographic orientation intensity is also reduced dramatically (maximum from 12.65 to 2.59) which will lead to more isotropic mechanical properties. Figure 8b does not indicate any regions of partially unmelted powder as seen in Fig. 7b It is worth noting, Fig. 7b was produced at 1200 mm/min compared with Fig. 8b being produced at 950 mm/min which may indicate where the threshold of printability for these Ti alloys lies. Grain refinement was achieved alongside a homogenous melt, pointing towards the partially unmelted powders being a result of the process parameters, not the causation of grain refinement. These findings demonstrate significant potential for application in other titanium alloys where grain refinement is a desired objective.

## Conclusion

This paper employs a methodology in which build responses can be collected and accurately modelled from minimal experiments over a chosen process parameter range. This research shows time and resource efficient experimentation for applicable response modelling, with applications in process parameter optimization. From this methodology it has been shown that the use of GED as an input parameter is overly simplistic and modelling as a product of the constituent parameters; laser power, laser scanning speed and spot size produces much more accurate and useful results. For the experimental material, Ti–10Fe, models were fit to the responses of manufacturing layer height and average grain size.

- The layer height was most influenced by the laser scanning speed, then the spot size and least influenced by the laser power. An accurate predictive model for layer height was produced using an ANN. This breakthrough enables the user to set a layer height from chosen process parameters, avoiding iterative layer height setting. As a result, the production time for a 10 × 10 × 10 mm^3^ cube can be dramatically reduced, going from 12.5 min down to just 5.5 min, equating to more than a 50% reduction in production time.
- The average grain size varied between 38 and 133 μm due to the changing process parameters. An ANN and MLR were both accurate in modelling the response. The laser power and laser scanning speed were most influential on the average grain size response. By decreasing laser power and increasing the laser scanning speed, grain refinement is possible. This was applied to another α + β Ti alloy, reducing the average grain size from 119 to 68 μm and produced less crystallographic texture.

Both the MLR and ANN produced accurate models, however the ANNs showed benefit over the MLR models with marginally less error. Using the methods in tandem allowed the accurate modelling of the ANN coupled with insights from produced MLR equations. This methodology is anticipated to be adaptable for a wide range of materials and easily customizable for different manufacturing processes, including PBF-LB and PBF-EB. Beyond its efficiency in predicting optimal layer height, this approach has the potential to revolutionize the control of material grain size. Its applicability extends far beyond just one parameter, as it opens opportunities to explore other critical process factors, such as powder flow rate, and their impact on various responses.

### Supplementary Information

Supplementary Information.

## Data Availability

The datasets used and/or analysed during the current study available from the corresponding author on reasonable request.
